# The Hering Memorial Volume

**Published:** 1884-07

**Authors:** 


### The Hering Memorial Volume. Prepared by Drs. Rane, Knerr, and Mohr. Philadelphia, 1884.

This volume is designed to give expression to the gratitude of the homœopathic profession for the great life and work of Constantine Hering.

No eulogies can add to his fame; that rests on the grand life achievements of the man. Nevertheless, this biographical sketch and these addresses are but the mete and just expression of appreciation, admiration, and gratitude from those who are now reaping the benefit of the tireless and unselfish labors of our lamented leader. As a further evidence of this gratitude EVERY physician should purchase a copy. The homœopathist who does not possess this memorial volume should be ashamed to acknowledge it.

